# Brain cavernomas associated with en coup de sabre linear scleroderma: Two case reports

**DOI:** 10.1186/1546-0096-9-18

**Published:** 2011-07-29

**Authors:** Emily T Fain, Melissa Mannion, Elena Pope, Daniel W Young, Ronald M Laxer, Randy Q Cron

**Affiliations:** 1 Department of Pediatrics, Division of Rheumatology, University of Alabama at Birmingham, Birmingham, AL, USA; 2 Section of Dermatolgy, Division of Paediatric Medicine, Department of Paediatrics, The Hospital for Sick Children and University of Toronto, Toronto, ON, Canada; 3 Department of Radiology, Children's Hospital of Alabama, Birmingham, AL, USA; 4 Division of Rheumatology, Departments of Paediatrics and Medicine, The Hospital for Sick Children, University of Toronto, Toronto, ON, Canada

## Abstract

Linear scleroderma is a form of localized scleroderma that primarily affects the pediatric population. When it occurs on the scalp or forehead, it is termed "en coup de sabre". In the en coup de sabre subtype, many extracutaneous associations, mostly neurological, have been described. A patient with linear scleroderma en coup de sabre was noted to have ipsilateral brain cavernomas by magnetic resonance imaging. Using a worldwide pediatric rheumatology electronic list-serve, another patient with the same 2 conditions was identified. These two patients are reported in this study. Consideration of neuroimaging studies to disclose abnormal findings in patients with linear scleroderma en coup de sabre is important for potentially preventing and treating neurological manifestations associated with this condition.

## Background

Localized scleroderma encompasses the conditions of linear scleroderma (LS) (extremity and facial), plaque or circumscribed morphea, pansclerotic and generalized morphea. By definition, localized scleroderma involves the skin and underlying tissue. This is opposed to systemic scleroderma, which not only involves skin but includes internal damage to the lungs, gastrointestinal tract, and vascular system.

Linear scleroderma is a form of localized scleroderma that primarily affects the pediatric population. Up to two-thirds of patients given this diagnosis are under the age of 18, and males and females are equally affected [1,2]. The lesions are typically sclerotic and discolored. When these lesions occur on the scalp or forehead, they are termed "en coup de sabre" [3-6]. In the en coup de sabre subtype, extracutaneous associations, mostly neurological, have been described [7]. However, internal organ involvement beyond the central nervous system (CNS) is extremely rare.

Linear scleroderma en coup de sabre is typically ipsilateral and near the midline of the forehead with extension into the frontoparietal scalp. It can be associated with hair loss and significant sclerosis of the skin. Often en coup de sabre lesions coexist with Parry-Romberg Syndrome (PRS), which involves hemiatrophy of the subcutaneous tissue and bones of the face without skin or scalp involvement [3-5,8].

The most commonly described brain lesions in LS en coup de sabre are intracranial calcifications, which are characteristically ipsilateral to the skin lesions, and appear as hypointense white matter lesions on T2-weighted magnetic resonance imaging (MRI) [5,6,9]. Hyperintense white matter lesions on T2-weighted MRI are also commonly seen in these patients [10]. Other neuroimaging abnormalities described in LS en coup de sabre include cerebral hemiatrophy, hyperintensities in deep white and gray matter structures, nonspecific alterations in cerebral vasculature [11], and intracranial aneurysms [12].

Cerebral cavernomas are vascular malformations located in the central nervous system. They are present in approximately 1 in 200 patients and approximately 20% of cavernomas are related to an autosominal dominantly inherited familial disease. Cavernomas consist of vessels that lack smooth muscle support and are thus prone to rupture, which can cause hemorrhage, stroke, seizures, or even death [13]. To our knowledge, there have been no reports describing multiple cavernomas on the ipsilateral side of a patient with LS en coup de sabre. Herein, the diagnosis, treatment, and outcomes of two patients with LS en coup de sabre and ipsilateral cavernomas at two large academic institutions are discussed.

## Case Presentation

A patient with LS en coup de sabre at the Children's Hospital of Alabama was noted to have brain cavernomas by MRI. This child was one of 4 children with LS en coup de sabre seen, among 59 LS and morphea patients, over 10 years a this institution, and he was the only one of the 4 LS en coup de sabre children imaged with cavernomas by MRI. Using a worldwide pediatric rheumatology electronic list-serve, another patient at the The Hospital for Sick Children, Toronto, with the same two conditions was identified. This unique child was identified from among over 300 LS patients (~70 LS en coup de sabre patients) seen over a 20 year period in Toronto, and none prior with brain cavernomas. However, not all patients received neuroimaging. These two patients are reported in this study.

### Case #1

**Figure 1:**
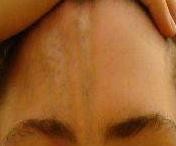
**En coup de sabre lesion**. A digital photographic image of the linear scleroderma/en coup de sabre lesion early in the course of therapy for case #1.

**Figure 2:**
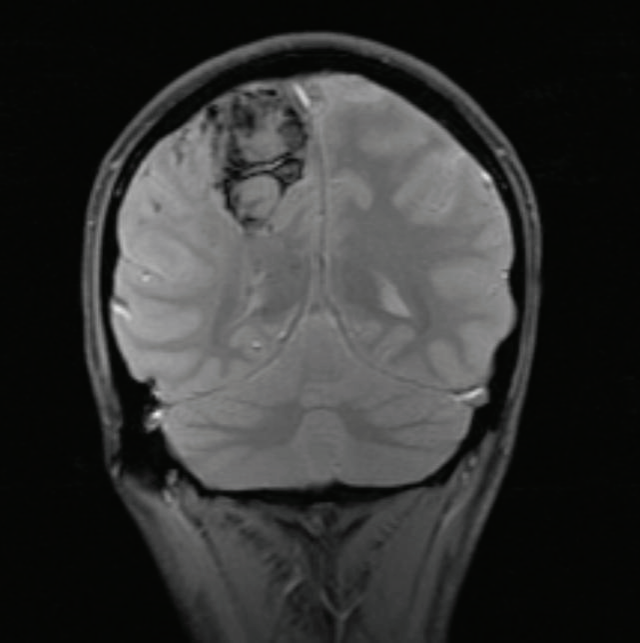
**Brain cavernomas**. Coronal cut MRI image of the brain cavernomas located ipsilateral and directly beneath the en coup de sabre linear scleroderma lesion of case #1.

A 13-year-old white male, with LS/PRS since age 3, at the Children's Hospital of Alabama was referred to a pediatric neurologist for the evaluation of two generalized tonic-clonic seizures initially treated with topiramate. There had been no preceding fever or trauma, although the patient noted a 6-month history of worsening headaches. An EEG was normal, but an outside MRI revealed effacement of sulci on the right side of his brain. In addition, there was diffuse white matter increased signal and T2 flair sequence on the right. Finally, MRI showed multiple right-sided low densities creating an echo sequence, likely representing microhemorrhage or calcification. Further diagnostic evaluation was declined, and he was started on topiramate 50 mg twice daily with no additional seizure activity noted. He was then referred to a pediatric rheumatologist for further evaluation and treatment. At initial evaluation 9 months later, an en coup de sabre lesion was noted near the midline on right side of the face, extending from nose to hairline and had become sunken per the family. The lesion was noted to be hypopigmented with violaceous borders. There was an approximately 1.5 cm in diameter and 0.5 cm in depth depression in the middle of his head near the apex and just to the right of the midline on the right side of the face (Figure 1). There were no other lesions noted on his body nor was he systemically ill. An ophthalmologic exam revealed no ocular inflammation. Upon presentation to rheumatology, since the lesion appeared to be getting worse and headaches and seizures were ongoing, the patient was initially treated with prednisone 40 mg twice daily for one month, tapered over the course of 2-3 months, and methotrexate 25 mg injected subcutaneously each week. Over the course of the next year the patient remained on methotrexate, with doses ranging from 12.5 to 25 mg weekly. On this treatment, the skin lesion improved (less violaceous and less indurated) significantly. The patient also remained seizure free during this time period. After approximately 1 year of successful treatment, the family chose to discontinue the methotrexate, and he remained off of consistent treatment for the next year. Approximately 23 months after his initial seizure the patient was noted to have a severe headache and the sudden onset of left-sided hemiparesis and parathesias. An MRI scan showed a 4 cm hemorrhage in the right frontoparietal region with mass effect, areas of increased white matter T2 signaling anterior to the lesion in the right temporal lobe, and areas of signal drop out in a punctuate fashion in many areas in the right hemisphere, most consistent with multiple right-sided cavernomas. However, other primary vascular lesions cannot be formally ruled out without biopsy. A concomitant MRI/MRA (MR angiogram) showed the same (Figure 2). Surgical intervention was declined, but there was ongoing concern for CNS inflammation, and the patient was admitted to the pediatric intensive care unit and started on topiramate, mycophenolate mofetil (MMF), and prednisone. The patient had residual focal neurological deficits, but otherwise did well and was discharged to home on MMF 1,500 mg twice daily, prednisone 40 mg daily, and topiramate 50 mg daily. The prednisone was weaned over the course of 3 months. The patient remained on MMF, and doses were gradually decreased to 1,500 mg daily over the course of 8 months. The skin lesion remained stable, but, the patient had a recurrent partial seizure 6 months after his hemorrhage, described as an episode of left arm and leg flexion lasting 3-4 minutes. There were no focal neurological changes, and the patient's topiramate was increased to 100 mg daily. The patient has had no subsequent seizures to this point. He was treated with extensive physical rehabilitation and made significant improvement, although his left lower extremity continues to have spasticity. On motor testing he does have mild left hemiparesis on his face, arm, and leg. It is most prominent in his arm, and it is more evident distally than proximally. He walks with a mild hemiparetic gait, and his right side strength remains good. Throughout the course of his two years of treatment at Children's Hospital of Alabama, the patient tested negative for an elevated erythrocyte sedimentation rate or C-reactive protein, anti-nuclear antibody, rheumatoid factor, Scleroderma-70 antibody, double-stranded DNA antibody, lupus anticoagulant, anti-neutrophil cytoplasmic antibody, cardiolipin antibody, and Sjogren syndrome A and B antibodies.

### Case #2

A 9 year-old-girl was referred to the Dermatology Clinic at The Hospital for Sick Children with a 6-month history of two facial lesions, one involving the right forehead and the second involving the right cheek. This began as a dime-sized lesion on the cheek and spread. There was no history of preceding infection, trauma, or travel. There were no headaches or ocular symptoms and she was otherwise well. Examination showed a linear lesion involving the right side of the forehead with a central white thickened sclerotic area surrounded by a violaceous rim. A similar plaque was located on the right cheek and had a similar appearance. The eye socket was spared. An ophthalmologic evaluation excluded uveitis. A diagnosis of facial morphea was made and treatment instituted with intravenous methylprednisolone, 30 mg/kg/dose, 3 daily doses given 4 weeks apart for 3 courses. In addition, prednisone 1 mg/kg initially in a twice-daily dose was started and weaned over 6 months. Methotrexate 15 mg/m^2 ^once a week was started together with 1 mg folic acid per day. On this treatment, the lesions improved significantly; there was softening, the violaceous discoloration disappeared, and the lesion became smaller. Because of some mild remaining firmness in the linear lesion, eight months later the methotrexate was increased to 20 mg once a week and given subcutaneously. Three years later, the methotrexate began to be weaned. She had no new lesions or progression of the initial lesions during her course of treatment. She had two minor elevations of her liver transaminases which resolved spontaneously, and several oral ulcers which resolved quickly while holding the methotrexate for one week. Two months after the initial wean of the methotrexate, the patient complained of numbness of her left hand and foot. She had an un-witnessed fall but then seemed to be well and attended school. Several hours later she had a secondarily generalized tonic-clonic seizure which began on the left side. It required diazepam for control. There had been no preceding fever or trauma. A general physical exam was normal. Ophthalmologic evaluation was normal. She was treated with phenytoin and dexamethasone. A CT scan demonstrated a 1.8 × 2.0 cm high density lesion in the right parietal lobe, most likely representing hemorrhage without significant enhancement. There was a moderate amount of surrounding vasogenic edema and no evidence of infarction. A concomitant MRI also revealed multiple smaller low signal lesions on gradient echo T2 imaging suggesting that the lesion most likely represented a cavernoma that had bled. An MRI done 9 days later revealed that the lesion had decreased dramatically in size and the edema had resolved. There were multiple additional smaller foci involving the right cerebral hemisphere likely representing calcification or hemosiderin due to other small cavernomas. The patient was discharged on carbamazepine, has remained seizure free, and has done extremely well. Her neurologic examination was normal and there were no cognitive deficits.

## Discussion

Multiple case studies have been published describing neurological dysfunction in associated with LS. Typically the skin lesion develops prior to the onset of neurological symptoms, although cases of the reverse have certainly been described [1]. A literature review done by Kister et al. found that the skin lesion preceded the onset of neurological symptoms by an average of several years (skin lesion appeared at age 9.5 years +/- 7.9, neurological symptoms onset at age 13.8 years +/- 10), although in 29% of the patients studied the two occurred within one year of each other [7]. Often times a patient with LS has been incidentally found to have abnormalities on neurological imaging, even when there are no neurological symptoms present [5,14]. With regards to the two patients described above, one patient's appearance of skin lesion and CNS symptoms was 10 years apart, while the other patient's was 3 years between. In both cases the skin lesion preceded the onset of neurologic symptoms.

Many case studies have described the association between LS and neurologic abnormalities. A case study in 2002 described a patient with LS and progressive atrophy of the ipsilateral cerebral hemisphere, not associated with overlying facial lesions [15]. Another in 2009 described a patient with refractory partial seizures prior to the development of a skin lesion on his head [16]. A ten-year old boy with PRS and progressive neurological deficit was found to have a giant intracranial aneurysm [10]. A case study in 1999 reported a patient who had de novo formation of a cavernoma three months after beginning immunosuppressive therapy with methotrexate; however, this patient was known to have a venous malformation [17]. Nevertheless, to the knowledge of the authors, these 2 cases represent the first time the finding of LS en coup de sabre with associated ipsilateral multiple brain cavernomas has been described. There is certainly a possibility that these two findings are unrelated and simply occurred by chance, but secondary to the ipsilateral nature of these two clinical findings, it is considerably more likely that there is in fact an association between the two.

The association between LS and associated neurological involvement on the ipsilateral side has been documented. A study of 55 neurologically symptomatic patients with LS showed that 78% had CNS lesions only on the side of the skin lesion [7]. Two similar case studies of neurologically asymptomatic patients with LS who received CNS imaging showed that 82-100% (n = 13) had exclusively ipsilateral lesions in the brain [18,19].

The pathogenic mechanisms behind neurological dysfunction associated with LS and PRS are unclear, although several theories have been proposed. Perhaps the most plausible and widely accepted explanation is the "neurovasculitis hypothesis" based on focal MRI findings and angiographic evidence of CNS vascular changes [20-22]. Many physicians believe that LS belongs in the collagen-vascular group of autoimmune diseases, such as discoid and systemic lupus erythematosus [23]. Alternatively, because neurologic abnormalities in LS en coup de sabre and PRS typically occur on the ipsilateral side, others believe that a defect in a common cell progenitor led to early malfunction affecting one side of the rostral tube during early neurological development [24]. Still others believe that an infectious source (such as *B. burgdorferi*) combined with predisposing genetic factors may be responsible for the etiology [3,4]. However, there does not appear to be an association with brain cavernomas and lupus or Lyme disease. Finally, it is interesting to speculate that factors leading to increased angiogenesis in the brain [25] may be related to increased angiogenesis in localized forms of scleroderma [26].

Neuroimaging studies should be considered in all patients with LS en coup de sabre at the time of diagnosis. In patients with any neurological signs or symptoms, these imaging studies become necessary [15]. Alternately, for patients with partial seizures, a thorough facial and scalp exam should be completed to look for skin changes associated with LS. Since neurological abnormalities can manifest themselves at differing points during the course of disease, it is important to have careful follow-up in patients with LS en coup de sabre as this may help direct theapy

Two different treatment approaches were employed for the cases reported herein. For patient #1, there was concern for ongoing inflammation so increased immunosuppression was employed. Dose reduction in MMF was eventually required secondary to diarrhea and weight loss. By contrast, the linear scleroderma in patient #2 was felt to be inactive and potentially unrelated to the cavernoma. Seizure management was the mainstay of therapy. Whether or not continued immunosppression would have helped prevent the CNS events reported herein remains unclear, but the risk benefit ratio of immunosuppression needs to be considered for each patient. In addition, studies in man and in rats suggest that immunosuppression can improve neurologic outcomes following aneurismal bleeds in the CNS [27,28]. Further study is warranted on the role of immunosuppressive therapy in preventing and treating CNS bleeds in individuals with CNS cavernomas.

## Conclusions

En coup de sabre LS has known ipsilateral CNS associations. Herein, we report the first associations with ipsilateral CNS cavernomas. In both cases presented, the cavernomas bled with notable clinical manifestations. At present, it is unclear whether these bleeds were associated with ongoing inflammation as part of the LS. Nevertheless, these cases highlight the importance of CNS imaging in children with en coup de sabre LS.

## Consent

Written informed consent was obtained from the patients (and the patients' respective parents) for publication of this case report. Institutional Review Board approval was obtained from the University of Alabama at Birmingham, and a waiver for case reports was approved from the University of Toronto.

## List of abbreviations used

CNS: central nervous system; CT: computerized tomography; LS: linear scleroderma; MRI: magnetic resonance imaging; PRS: Parry-Romberg Syndrome.

## Competing interests

The authors declare that they have no competing interests.

## Authors' contributions

EF and MM wrote the initial manuscript with contributions from EP. DY performed the analysis and interpretation of the radiological data. EP, RL and RC were directly involved in the patient care and participated in writing the manuscript and are responsible for its final editing. All authors read and approved the final manuscript.
